# Reduced Vancomycin Susceptibility Found in Methicillin-Resistant and Methicillin-Sensitive *Staphylococcus aureus* Clinical Isolates in Northeast China

**DOI:** 10.1371/journal.pone.0073300

**Published:** 2013-09-12

**Authors:** Jian Hu, Xiao Xue Ma, Yuan Tian, Long Pang, Long Zhu Cui, Hong Shang

**Affiliations:** 1 Department of Medical Microbiology and Parasitology, College of Basic Medical Sciences, China Medical University, Shenyang, People's Republic of China; 2 Research Center for Anti-infectious Drugs, Kitasato University, Sagamihara, Kanagawa, Japan; 3 Department of Clinical Laboratory, First Affiliated Hospital of China Medical University, Shenyang, People's Republic of China; 4 Department of Clinical Laboratory, Yixing Hospital of Traditional Chinese Medicine, Yixing, Jiangsu, People's Republic of China; Rockefeller University, United States of America

## Abstract

**Background:**

Strains of *Staphylococcus aureus* with an intermediate level of resistance to vancomycin (vancomycin-intermediate *S. aureus*, or VISA) or which contain subpopulations of mixed susceptibility (heterogeneous VISA, or hVISA) have been reported worldwide. However, the prevalence of VISA and hVISA infections in Northeast China is unknown. From 2007 through 2010, we surveyed the vancomycin susceptibility of methicillin-resistant and methicillin-sensitive *S. aureus* (MRSA and MSSA, respectively) clinical isolates in Northeast China.

**Methods:**

*S. aureus* clinical isolates (369 MRSA and 388 MSSA) were screened for hVISA and VISA on brain heart infusion agar containing 3 μg/mL vancomycin, and their identity confirmed using a modified population analysis profile-area under the curve method and broth microdilution. All hVISA and VISA isolates were characterized genotypically and phenotypically.

**Results:**

Ten percent and 0.5 percent of the isolates were hVISA and VISA, respectively. The proportion of hVISA among MSSA isolates for the entire study period was 4.1%, but increased significantly year-by-year, from 1.2% in 2007 to 7.2% in 2010. The predominant sources of hVISA and VISA isolates were sputum (56.3%), pus (18.8%), and blood (8.8%). Molecular typing of hVISA and VISA strains revealed that, taken together, 80% contained the accessory gene regulator (*agr*) group II, and of these, 85.7% of the MR-hVISA and MR-VISA strains were staphylococcal cassette chromosome *mec* (SCC*mec*) type II. The adherence ability of all hVISA and VISA strains was reduced compared with that of vancomycin-susceptible strains, shown by biofilm assay.

**Conclusions:**

The percentage of hVISA strains was high and increased each year. The proportion of hVISA among MSSA specifically also increased significantly each year. In isolates collected from diverse infection sites, hVISA and VISA strains were found predominantly in sputum, pus, and blood, in descending order. Testing for vancomycin susceptibility should include both MRSA and MSSA isolates collected from different clinical sites.

## Introduction

*Staphylococcus aureus* is a ubiquitous bacterium responsible for both community-associated and hospital-acquired infections, which range in severity from non-pathogenic to life threatening [1]. These infections, especially those due to methicillin-resistant *S. aureus* (MRSA), have been treated for more than a half-century primarily with the glycopeptide antibiotic vancomycin. Unfortunately, increased incidence of nosocomial MRSA infections has led worldwide to the development of *S. aureus* strains with varying degrees of resistance to vancomycin [2]–[6], first reported in 1997 [7], [8].

Reduced susceptibility to vancomycin in *S. aureus* is complex and difficult to detect in clinical microbiology laboratories [9], [10]. In January 2006, to improve the correlation between *in vitro* susceptibility and clinical response, the Clinical and Laboratory Standards Institute (CLSI; formerly NCCLS) redefined resistance breakpoints for vancomycin against *S. aureus* [9]. The current CLSI guidelines suggest that *S. aureus* strains should be categorized as susceptible, intermediate, or resistant when the vancomycin minimum inhibitory concentration (MIC) is ≤2 μg/mL, 4 to 8 μg/mL, or ≥16 μg/mL, respectively.

Clinical treatment failures with vancomycin have been linked to strains whose susceptibility to vancomycin falls within the intermediate MIC range (vancomycin-intermediate *S. aureus*, or VISA) and also strains that contain subpopulations of mixed susceptibility (heterogeneous VISA, or hVISA). To characterize VISA strains, determinations of vancomycin broth microdilution MICs performed in accordance with CLSI criteria should be used [9]–[11]. However, the breakpoints that differentiate hVISA strains from vancomycin-susceptible *S. aureus* (VSSA) have not been established. [10], [11]. While the vancomycin MICs of hVISA fall within the susceptible range when tested by routine methods, these strains stably produce subsets of cells (typically one organism per 10^5^ to 10^6^) in the intermediate range; they are assumed precursors of VISA [8].

A variety of screening assays have been developed to detect hVISA [4], [8], [12], [13], but to date there is no standardized approach that is convenient and reliable. The gold standard for confirmation of hVISA is the population analysis profile-area under the curve (PAP-AUC) method, which is time-consuming, labor-intensive, and costly for routine clinical laboratories [10], [14]. More recently, a novel agar with 3 μg/mL vancomycin was advocated for screening vancomycin non-susceptible *S. aureus* isolates, with a reported 100% sensitivity and 65% specificity based on evaluation of 100 *S. aureus* isolates (55 VSSA and 45 VISA) [15]. We also evaluated a screening procedure using brain heart infusion agar containing 3 μg/mL vancomycin (BHIA-3V) for detection of hVISA with PAP-AUC, and found that this method had 100% sensitivity to hVISA (data not shown). In light of the excellent sensitivity of the BHIA-3V screening procedure for detection of hVISA and VISA [15]–[17], in the present study we employed this approach to screen *S. aureus* isolates with decreased susceptibility to vancomycin.

Since the first descriptions of hVISA (Mu3) and VISA (Mu50) strains by Hiramatsu *et al*. from Japan in 1997 [7], [8], clinical isolates of *S. aureus* with decreased susceptibility to vancomycin have been reported in many countries, including the United States [2], [18], Canada [6], China [4], Japan [7], [8], [19], South Korea [20], Australia [21], United Kingdom [3], [14], Italy [5], and France [22], and their occurrence has become a major concern throughout the world. However, although China accounts for one-sixth of the world's population, there are only a few reports from mainland China regarding hVISA or VISA isolates specifically [4], [20], [23]. In one study, 1012 vancomycin-susceptible MRSA isolates obtained from the years 2005 to 2007 in 14 cities in China were investigated, and the estimated prevalence of hVISA was 13% to 16% [4]. With this in mind, we hypothesized that the incidence rate of hVISA and VISA infections might be high in hospitals located in Northeast China, one of the most populated regions in the world. Moreover, although hVISA and VISA have been reported predominately in MRSA strains [2]–[8], they are also present among MSSA, suggesting the need to screen both MRSA and MSSA isolates if a true prevalence is to be determined [24], [25].

Hence, we began the present study, the first such in Northeast China, by investigating the prevalence of hVISA and VISA strains among clinical isolates of *S. aureus* collected in two large teaching hospitals over a 4-year period. Our study may provide incentive for a larger-scale investigation and prevention program, not only in China but also within all international biomedical and epidemiological communities.

## Materials and Methods

### Bacterial strains and culture conditions

From January 2007 to January 2011, a total of 757 consecutive *S. aureus* isolates were collected at the First Affiliated Hospital (2249 beds) and Second Affiliated Hospital (4818 beds) of China Medical University in Shenyang, the capital city of one of the three provinces of Northeast China. These two hospitals are large tertiary hospitals with 237,000 annual admissions (100,000 and 137,000 respectively); 13,280 staff personnel (4000 and 9280); and 57,799 students; and can be considered representative of large metropolitan hospitals in China, serving patients mainly from all over Northeast China.

One *S. aureus* isolate from each patient was included in this retrospective study. The Medical Ethics Committee of China Medical University, Shenyang, China (Chairperson Prof Qun Zhao) granted ethical approval for this study on 12 February 2011 (Ethical Committee No. 11021207). Each patient who was enrolled in this study signed the informed consent form. Isolates of *S. aureus* were identified with the positive tube coagulase test and a VITEK 2 automated system (bioMérieux, France). MRSA isolates were confirmed using the cefoxitin disk diffusion method and correlated with the presence of the *mecA* gene by PCR [26], [27]. All strains were routinely stored at −80°C in brain heart infusion (BHI) broth (Becton Dickinson, Sparks, MD, USA) containing 20% (v/v) glycerol, and 48 h prior to testing were subcultured twice onto Columbia agar containing 5% sheep blood (Oxoid, Basingstoke, UK).

### Detection of hVISA and VISA

All *S. aureus* isolates were screened for hVISA and VISA strains on BHIA-3V plates as previously described [15], [16]. The culture was considered positive if there was growth of one or more colonies after 48 h. The agar plates were prepared in-house daily and the screening tests were performed in duplicate. Isolates which displayed a hVISA/VISA profile on the BHIA-3V screening plates were further confirmed by the PAP-AUC approach using the technique of Wootton *et al*. [14]. An isolate was considered hVISA if the ratio of the AUC of the test strain to that of Mu3 was ≥0.9; an isolate with a ratio of <0.9 was defined as VSSA. The VSSA strain ATCC 29213 was used as a negative control. The hVISA strain Mu3 (ATCC 700698) and VISA strain Mu50 (ATCC 700699) were used as positive controls. The results from each experiment were recorded only when positive and negative controls were confirmed.

### Antimicrobial susceptibility testing and reagents

The routine disk diffusion method with antibiotic disks (Oxoid) was used to determine the susceptibility of all screen-positive isolates to oxacillin, clindamycin, gentamicin, chloramphenicol, trimethoprim-sulfamethoxazole (TMP-SMX), erythromycin, ciprofloxacin, levofloxacin, tetracycline, and rifampin in accordance with the CLSI guidelines [28]. In addition, MICs of vancomycin, teicoplanin, linezolid, daptomycin, ceftobiprole, and tigecycline were determined by broth microdilution using cation-supplemented Mueller-Hinton broth (Oxoid), as recommended by the CLSI guidelines [28].

The results were independently interpreted by two investigators. Isolates for which the vancomycin MIC was 4 to 8 μg/mL were considered VISA, in accordance with the criteria of the CLSI [11]. Vancomycin, teicoplanin, and daptomycin were obtained from Sigma Chemical, St. Louis, MO, USA; linezolid and tigecycline were obtained from Pfizer, NewYork, NY, USA.

### DNA extraction and molecular typing

DNA was prepared by the small-scale phenol extraction method, as described previously [29]. The DNA was used as the template in all PCRs described below. All hVISA and VISA isolates identified were analyzed by staphylococcal protein A (*spa*) typing as previously described [30]. One strain representative of each major *spa* type was further characterized by multilocus sequence typing (MLST) [31], [32]. All hVISA and VISA isolates were analyzed by accessory gene regulator (*agr*) grouping using multiplex PCR in accordance with the published procedure [33]. All methicillin-resistant hVISA (MR-hVISA) and methicillin-resistant VISA (MR-VISA) isolates were typed by staphylococcal cassette chromosome *mec* (SCC*mec*) using multiplex PCR as previously described [34]. All hVISA and VISA isolates were detected for the Panton-Valentine leukocidin (PVL) toxin gene in accordance with the protocols of prior reports [35], [36].

### Delta-hemolysin expression, biofilm assay, and autolysis assay

All screen-positive isolates by BHIA-3V were subjected to delta-hemolysin expression, biofilm assay, and autolysis assay. The function of the *agr* gene cluster was determined by delta-hemolysin production as described by Sakoulas *et al*. [37]. The ability of *S. aureus* to adhere to a polystyrene microtiter plate was evaluated as described previously [21], [37]. The absorbance was measured at 570 nm, and each assay was performed five times. Autolysis of *S. aureus* in Triton X-100 was performed as described previously [21]. The OD600 was measured each hour for 6 h, and each assay was performed five times.

### Statistical analyses

All statistical analyses were performed using SPSS for Windows, version 12.0 (SPSS, Chicago, IL, USA). Categorical variables were compared using the chi-squared (χ^2^) or Fisher's exact probability tests, including prevalence rates and antimicrobial susceptibility of hVISA/VISA. A p-value of <0.05 was considered statistically significant.

## Results

### Prevalence rates of hVISA and VISA isolates in clinical specimens

In total, 757 non-duplicate *S. aureus* isolates were investigated in this study. The proportion of MRSA and MSSA isolates were 48.7% (369/757) and 51.3% (388/757), respectively (Table 1). The number of isolates tested in each year were: 2007, n = 171 (88 MRSA and 83 MSSA); 2008, n = 183 (89 MRSA and 94 MSSA); 2009, n = 197 (97 MRSA and 100 MSSA) and 2010, n = 206 (95 MRSA and 111 MSSA). The isolates were recovered from sputum (n = 249), pus (n = 208), blood (n = 137), secretions (n = 84), drainage (n = 35), wounds (n = 23), or other (n = 21).

**Table 1:** All hVISA and VISA isolates identified from different clinical specimens a.

			Confirmed by PAP-AUC c		Isolates from different clinical specimens (n = 80)					
	Isolates	BHIA-3V+ b	hVISA	VISA	Sputum	Pus	Blood	Secretion	Drainage	Other
MRSA	369 (48.7)	120 (32.5)	60 (16.3)	3 (0.8)	43 (53.8)	6 (7.5)	6 (7.5)	4 (5)	2 (2.5)	2 (2.5)
MSSA	388 (51.3)	89 (22.9)	16 (4.1)	1 (0.3)	2 (2.5)	9 (11.3)	1 (1.3)	1 (1.3)	1 (1.3)	3 (3.8)
Total	757	209 (27.6)	76 (10.0)	4 (0.5)	45 (56.3)^d^	15 (18.8)	7 (8.8)	5 (6.3)	3 (3.8)	5 (6.3)

a All data are presented as number (%).

b BHIA-3V is the brain heart infusion agar containing 3 μg/mL vancomycin; 757 isolates were screened for hVISA and VISA on BHIA-3V plates.

c PAP-AUC is the population analysis profile-area under the curve; 209 screen-positive isolates were further confirmed by PAP-AUC.

d Prevalence of hVISA and VISA isolated from sputum versus other specimens (p<0.001).

Among the 757 isolates, 209 (120 MRSA and 89 MSSA) grew on BHIA-3V screening plates within 48 h (i.e., screen-positive isolates). Seventy-six (60 MRSA and 16 MSSA) of the 209 isolates were confirmed as hVISA via the PAP-AUC approach, and four (3 MRSA and 1 MSSA) were identified as VISA with the broth microdilution in accordance with the CLSI criteria.

Accordingly, the prevalence rates of hVISA and VISA among all *S. aureus* isolates from different specimens were 10.0% (76/757) and 0.5% (4/757), respectively. In particular, the hVISA prevalence was higher in MRSA strains (60/369, 16.3%) compared with MSSA (16/388, 4.1%; p<0.001). In addition, the predominant sources of hVISA and VISA isolates in decreasing order were sputum (n = 45, 56.3%; p<0.001), pus (n = 15, 18.8%), blood (n = 7, 8.8%), secretions (n = 5, 6.3%), drainage (n = 3, 3.8%), and other (n = 5, 6.3%).

In the year 2007, the percentage of hVISA was 8.2% (13 MR-hVISA, 7.6%; 1 methicillin-sensitive hVISA [MS-hVISA], 0.6%; p = 0.001). In 2008, 2009, and 2010, the percentages of hVISA were 9.3% (15 MR-hVISA, 8.2%; 2 MS-hVISA, 1.1%; p = 0.001), 10.6% (16 MR-hVISA, 8.1%; 5 MS-hVISA, 2.5%; p = 0.009), and 11.7% (16 MR-hVISA, 7.8%; 8 MS-hVISA, 3.9%; p = 0.032), respectively. Of note, the proportion of hVISA among MSSA isolates was 4.1% and increased from 1.2% (1/83) in 2007 to 7.2% (8/111) in 2010. Thus during the four year period examined, hVISA increased gradually year-by-year, and the proportion of MS-hVISA increased rapidly.

### Antimicrobial susceptibility

Antimicrobial activity of 15 antimicrobial agents against 209 screen-positive isolates (129 VSSA, 76 hVISA, and 4 VISA) *in vitro* showed that all these strains were susceptible to linezolid, daptomycin, and tigecycline (Table 2). MR-hVISA and MR-VISA strains were more resistant to several non-β-lactam antibiotics than were MR-VSSA strains, including chloramphenicol, TMP-SMX, and rifampin. Furthermore, MS-hVISA and MS-VISA strains were more likely to be resistant to all the antibiotics tested than were MS-VSSA strains.

**Table 2:** Antimicrobial activity of 15 antimicrobial agents against 209 screen-positive isolates *in vitro*.

		VSSA isolates, n (%)			hVISA/VISA isolates, n (%)			
		MRSA a	MSSA *^b^*	Total *^c^*	MRSA *^d^*	MSSA *^e^*	Total *^f^*	p-value *^g^*
Vancomycin MIC	0.5 μg/mL	10 (17.5)	16 (22.2)	26 (20.2)	0 (0)	0 (0)	0 (0)	
	1.0 μg/mL	25 (43.9)	29 (40.3)	54 (41.9)	19 (30.2)	2 (11.8)	21 (26.3)	
	2.0 μg/mL	22 (38.6)	27 (37.5)	49 (38.0)	41 (65.1)	14 (82.4)	55 (68.8)	
	4.0 μg/mL	0 (0)	0 (0)	0 (0)	3 (4.8)	1 (5.9)	4 (5)	
Teicoplanin MIC	1 μg/mL	17 (29.8)	33 (45.8)	50 (38.8)	2 (3.2)	2 (11.8)	4 (5)	
	2 μg/mL	24 (42.1)	25 (34.7)	49 (38.0)	18 (28.6)	8 (47.1)	26 (32.5)	
	4 μg/mL	13 (22.8)	12 (16.7)	25 (19.4)	31 (49.2)	5 (29.4)	36 (45.0)	
	8 μg/mL	3 (5.3)	2 (2.8)	5 (3.9)	11 (17.5)	2 (11.8)	13 (16.3)	
	16 μg/mL	0 (0)	0 (0)	0 (0)	1 (1.6)	0 (0)	1 (1.3)	
Susceptibility	Oxacillin	0 (0)	72 (100)	72 (55.8)	0 (0)	17 (100)	17 (21.3)	
	Clindamycin	5 (8.8)	40 (55.6)	45 (34.9)	0 (0)	0 (0)	0 (0)	<0.001
	Gentamicin	2 (3.5)	52 (72.2)	54 (41.9)	0 (0)	0 (0)	0 (0)	<0.001
	Chloramphenicol	44 (77.2)	70 (97.2)	114 (88.4)	30 (47.6)	4 (23.5)	34 (42.5)	<0.001
	TMP-SMX *^h^*	47 (82.5)	71 (98.6)	118 (91.5)	34 (54.0)	9 (52.9)	43 (53.8)	<0.001
	Erythromycin	1 (1.8)	6 (8.3)	7 (5.4)	0 (0)	0 (0)	0 (0)	0.085
	Ciprofloxacin	1 (1.8)	66 (91.7)	67 (51.9)	0 (0)	14 (82.4)	14 (17.5)	<0.001
	Levofloxacin	1 (1.8)	66 (91.7)	67 (51.9)	0 (0)	14 (82.4)	14 (17.5)	<0.001
	Tetracycline	2 (3.5)	59 (81.9)	61 (47.3)	0 (0)	12 (70.6)	12 (15)	<0.001
	Rifampin	39 (68.4)	72 (100)	111 (86.0)	25 (39.7)	14 (82.4)	39 (48.8)	<0.001
	Linezolid	57 (100)	72 (100)	129 (100)	63 (100)	17 (100)	80 (100)	
	Daptomycin	57 (100)	72 (100)	129 (100)	63 (100)	17 (100)	80 (100)	
	Tigecycline	57 (100)	72 (100)	129 (100)	63 (100)	17 (100)	80 (100)	

a n = 57; *^b^* n = 72; *^c^* n = 129; *^d^* n = 63; *^e^* n = 17; *^f^* n = 80; *^g^* No. of total VSSA isolates versus no. of total hVISA/VISA isolates; *^h^* TMP-SMX, trimethoprim-sulfamethoxazole.

The vancomycin MIC range for VSSA was 0.5 to 4 μg/mL, for hVISA was 1 to 2 μg/mL, and for VISA was 4 to 4 μg/mL (Table 3). The number of tested isolates with vancomycin MICs of 0.5, 1, 2, and 4 μg/mL were 26 (12.4%), 75 (35.9%), 104 (49.8%), and 4 (1.9%), respectively, as determined by broth microdilution. MIC_50/90_ of VSSA, hVISA, and VISA were 1/2, 2/2, and 4/4, respectively.

**Table 3:** MIC_50/90_ determined by CLSI broth microdilution and the prevalence of *agr*-dysfunction.

	Screen-positive *S. aureus* a	VSSA *^b^*	hVISA *^c^*	VISA *^d^*
Vancomycin range (μg/mL)	0.5–4	0.5–4	1–2	4–4
Vancomycin MIC_50/90_	2/2	1/2	2/2	4/4
Teicoplanin range (μg/mL)	1–16	1–8	1–8	4–16
Teicoplanin MIC_50/90_	2/4	2/4	4/8	8/16
No. (%) of *agr* -dysfunction	99 (47.4)	29 (22.5)	66 (86.8)	4 (100) *^e^*

a n = 209; *^b^* n = 129; *^c^* n = 76; *^d^* n = 4; *^e^* Prevalence of *agr* dysfunction in hVISA/VISA isolates versus VSSA isolates (p<0.001).

The teicoplanin MIC range for VSSA was 1 to 8 μg/mL, for hVISA was 1 to 8 μg/mL, and for VISA was 4 to 16 μg/mL. The number of tested isolates with teicoplanin MICs of 1, 2, 4, 8, and 16 μg/mL were 54 (25.8%), 75 (35.9%), 61 (29.2%), 18 (8.6%), and 1 (0.5%), respectively. MIC_50/90_ of VSSA, hVISA, and VISA were 2/4, 4/8, and 8/16, respectively.

The percentage of hVISA isolates with a vancomycin MIC of 2 μg/mL (55/76, 72.4%) was significantly higher than the percentage of VSSA isolates with the same MIC (49/129, 38.0%; p<0.001).

### *Spa* typing, MLST analysis, SCC*mec* typing, PVL gene detection, *agr* grouping and delta-hemolysin expression

Sequence analysis of the PCR products of the *spa* gene revealed 12 *spa* types (t002, t030, t037, t045, t105, t437, t034, t163, t189, t386, t548, and t2592) in the 80 hVISA/VISA isolates (Table 4). The most frequently encountered *spa* types were t002 (59/80, 73.8%), t030 (6/80, 7.5%), t037 (2/80, 2.5%), t045 (2/80, 2.5%), t105 (2/80, 2.5%), t386 (2/80, 2.5%), and t437 (2/80, 2.5%). One isolate each was identified for the five remaining *spa* types.

**Table 4:** Molecular characteristics of all hVISA and VISA isolates.

			*agr* group (n)				SCC*mec* type (n)						
hVISA/VISA (n)	*spa* type (n)	MLST-CC	I	II	III	IV	I	II	III	IV	V	NT *	PVL+
MR-hVISA (60)	t002 (47)	ST5-CC5		47				47					
	t030 (4)	ST239-CC8	3			1			4				
	t037 (2)	ST239-CC8	2					2					
	t105 (2)	ST5-CC5		2				1				1	
	t045 (2)	ST5-CC5		2							2		
	t437 (1)	ST59-CC59	1							1			
	t189 (1)	ST188-CC1	1						1				
	t548 (1)	ST5-CC5		1				1					
MS-hVISA (16)	t002 (8)	ST5-CC5		8									
	t030 (2)	ST239-CC8	2										
	t386 (2)	ST1-CC1											1
	t437 (1)	ST59-CC59	1		2								
	t034 (1)	ST398-CC398	1										1
	t163 (1)	ST59-CC59	1										
	t2592 (1)	ST88-CC88	1										
MR-VISA (3)	t002 (3)	ST5-CC5		3									
MS-VISA (1)	t002 (1)	ST5-CC5		1				3					

* NT, could not be typed.

The most frequent MLST type was ST5 (64/80, 80%), followed by ST239 (8/80, 10%), ST59 (3/80, 3.8%), and ST1 (2/80, 2.5%). ST5, ST239, and ST59 were found in both MRSA and MSSA isolates.

SCC*mec* types were identified in 63 MR-hVISA and MR-VISA isolates. Among them, the predominant type was SCC*mec* type II (54/63, 85.7%), followed by SCC*mec* type III (5/63, 7.9%), SCC*mec* type V (2/63, 3.2%), and SCC*mec* type IV (1/63, 1.6%). One MR-hVISA isolate could not be assigned to any SCC*mec* type using the established protocols.

PVL genes were found in one MR-hVISA strain and 2 MS-hVISA strains. One MR-hVISA strain was PVL-positive, belonging to ST59-MRSA-IV.

Regarding *agr* groups, *agr* group II was dominant (64/80, 80%), followed by *agr* group I (13/80, 16.3%), *agr* group III (2/80, 2.5%), and *agr* group IV (1/80, 1.3%). In addition, the expression of delta-hemolysin was evaluated for the 209 screen-positive isolates (129 VSSA, 76 hVISA, and 4 VISA). Overall, the percentages of *agr* dysfunctional phenotypes in VSSA, hVISA, and VISA were 22.5% (29/129), 86.8% (66/76), and 100% (4/4), respectively (Table 3). The prevalence of the *agr* dysfunctional phenotype was significantly higher in hVISA/VISA strains compared with that of the VSSA (p<0.001).

### Biofilm assay

**Figure 1:**
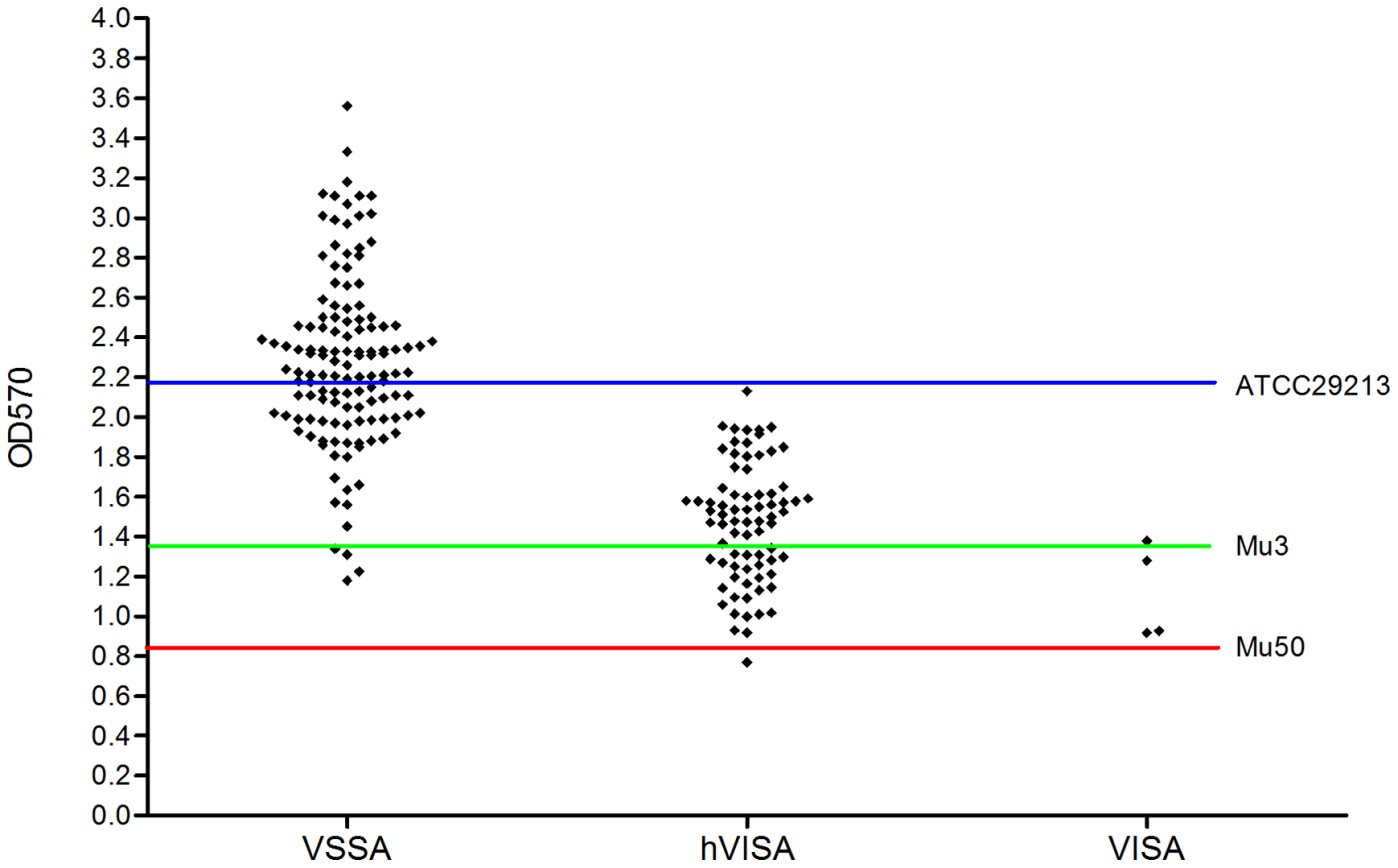
Quantification of biofilm formation was determined by crystal violet staining and read as an OD value. Each rhombus represents the mean OD value of one strain from five independent experiments. The ODs of most VSSA isolates (77/129, 59.7%) scattered above that of ATCC 29213 (the blue line; OD_570_ = 2.180), and the ODs of the majority of hVISA isolates (48/76, 63.2%) scattered between those of ATCC29213 and Mu3 (the green line; OD_570_ = 1.350). On the other hand, the ODs of the four VISA isolates scattered between those of Mu3 and Mu50 (the red line; OD_570_ = 0.848).

We performed the biofilm assay to compare the ability of the 209 screen-positive isolates (129 VSSA, 76 hVISA, and 4 VISA) to adhere to 96-well polystyrene microtiter plates (Figure 1). The adherence ability of these isolates is reflected by mean optical density values (ODs), which ranged overall from 0.769 to 3.560. The mean ODs for the VSSA, hVISA, and VISA isolates were 2.283 (range 1.180 to 3.560), 1.469 (range 0.769 to 2.129), and 1.125 (range 0.917 to 1.377), respectively. The ODs of most VSSA isolates (77/129, 59.7%) scattered above that of ATCC 29213 (the negative control), and the ODs of the majority of hVISA isolates (48/76, 63.2%) scattered between those of ATCC29213 and Mu3 (a positive control; Figure 1). On the other hand, the ODs of the four VISA isolates scattered between those of Mu3 and Mu50 (a positive control).

### Autolysis assay

**Figure 2:**
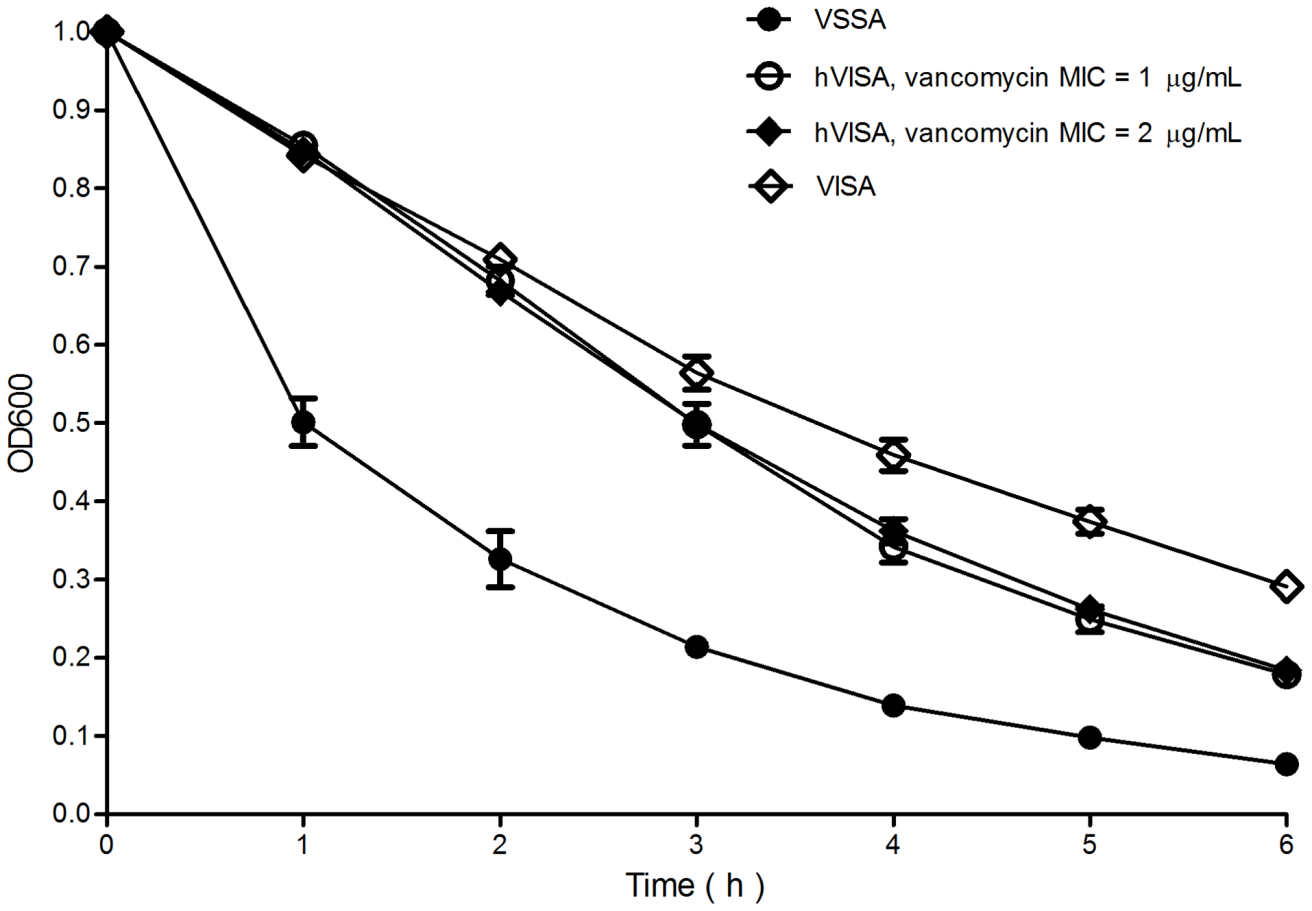
Autolysis assay results for 129 VSSA, 76 hVISA, and 4 VISA isolates. The results are expressed as the mean OD values of five independent experiments. Black circles indicate the mean OD values of 129 VSSA by hour; white circles and black rhombuses indicate the mean OD values of 21 hVISA with a vancomycin MIC of 1 μg/mL and the mean OD values of 55 hVISA with a vancomycin MIC of 2 μg/mL by hour, respectively; white rhombuses indicate the mean OD values of 4 VISA by hour.

We tested 209 screen-positive isolates (129 VSSA, 76 hVISA, and 4 VISA isolates) for autolytic activity and found that the autolytic activity of hVISA and VISA isolates was significantly less than that of VSSA isolates (Figure 2). No significant difference in autolytic activity was noted between hVISA isolates and hVISA isolates, which have a vancomycin MIC of 1 μg/mL and 2 μg/mL, respectively.

## Discussion

In the present study, during a four-year period (2007–2010) we surveyed 757 non-repetitive *S. aureus* isolates to determine the prevalence of hVISA and VISA. The data showed that in our hospitals in Northeast China, VISA clinical isolates were rare (0.5%), while the overall prevalence of hVISA (in MRSA and MSSA combined) was relatively high (10.0%). Furthermore, hVISA increased gradually during the four-year period examined, from 8.2% in 2007 to 11.7% in 2010. The high percentage and consistent increase of hVISA suggests a high potential for the development of complete drug resistance. We also found that the occurrence of hVISA was much higher (16.3%) in MRSA strains, which is similar to the results obtained by Sun *et al*. [4].

In an Asian surveillance study conducted in 2004 [20], a total of 1357 MRSA isolates obtained from 1997 to 2000 were investigated, and the hVISA rate was 4.3% (ranging from 0 to 8.2% among 12 countries). Additionally, in a systematic review published in 2011 of 43 epidemiological studies [38], the overall hVISA frequency in MRSA isolates was 1.3%, with a wide range from 0 to 73.7%. The reason for such a discrepancy in prevalence could be attributed to several factors, including differences in test strategies, geographic regions, and study populations.

The majority of reported hVISA and VISA isolates evolved from MRSA strains [2]–[8], yet in the present study we found that the proportion of hVISA among MSSA isolates was 4.1% and increased from 1.2% in 2007 to 7.2% in 2010, a 6-fold increase in four years. Liu and Chambers [24] in 2003 summarized data from 14 previous studies, and found that the prevalence of hVISA in 1868 MSSA isolates was 0.05%. Additionally, a study at one French hospital in 2006 which screened 2300 *S. aureus* isolates using a three-step approach, revealed that seven (0.3%) of these isolates were MS-hVISA strains [22]. In our study we found a significantly higher percentage of MS-hVISA, which increased rapidly each year. Hence, we believe that it is as important to detect reduced vancomycin susceptibility in MSSA isolates as in MRSA, and there is an alarming need to pay attention to the MS-hVISA population.

Most previous studies have focused on hVISA and VISA strains isolated from blood [2]–[6], whereas we found in the present study that hVISA and VISA strains were identified from diverse infection sites, and the predominant source of hVISA and VISA isolates was sputum (56.3%, p<0.001), followed by pus (18.8%), blood (8.8%), secretions 6.3%), drainage (3.8%), and other (6.3%). Therefore, in an effort to prevent the emergence and spread of vancomycin resistance, we recommend that *S. aureus* isolates from diverse clinical sites should be included when testing for reduced vancomycin susceptibility.

The antimicrobial susceptibility assay revealed all hVISA and VISA isolates were fully susceptible to the three alternative antistaphylococcal agents (linezolid, daptomycin, and tigecycline) in *vitro*. In addition, hVISA and VISA isolates differed from VSSA isolates by an overall higher frequency of drug resistance to multiple antibiotics, including clindamycin, chloramphenicol, TMP-SMX, and tetracycline. Interestingly, hVISA/VISA strains were significantly more likely to be resistant than VSSA strains to rifampin (51.3% compared with 14.0%, p<0.001). A previous study found that rifampin resistance developed more frequently in patients with hVISA than in those with VSSA during treatment of bacteremia [39], which suggested that rifampicin resistance was independently associated with the hVISA phenotype. Moreover, it was clearly demonstrated that hVISA strains tend to have higher vancomycin MICs within the susceptible range. A study by Leonard *et al*. in 2009, which evaluated a new epsilometer test (Etest) method for detection of hVISA using PAP-AUC, found that 49% of the hVISA strains had a vancomycin MIC of 2 μg/mL [40]. In the present study, no hVISA isolate with MIC <1 μg/mL was detected, and 72.4% (55/76) of the hVISA strains had a vancomycin MIC  = 2 μg/mL, as determined by broth microdilution. This suggested that we should closely monitor the effect of vancomycin treatment to avoid therapy failures when treating *S. aureus* infections with a vancomycin MIC ∼2 μg/mL.

The results of molecular typing showed that the hVISA phenotype was prevalent among SCC*mec* type II and *agr* group II isolates. Previous studies have indicated that *S. aureus* isolates exhibiting reduced vancomycin susceptibility were more likely to harbor SCC*mec* type II [41], [42]. Furthermore, SCC*mec* type II has been associated with increased mortality in MRSA [42], [43]. On the other hand, *agr* group II has been frequently associated with reduced vancomycin susceptibility, as well as vancomycin treatment failure [37], [44]. Given this, further understanding of factors that determine virulence will be of importance for preventing the dissemination of *S. aureus* with reduced vancomycin susceptibility and treating the related infections. We also note that the majority (80%) of hVISA and VISA isolates were from clonal complex 5, in particular ST5 (CC5), which is consistent with prior evidence [7], [8], [45]. Of interest, we found a ST398-MSSA isolate identified as a hVISA by PAP-AUC with vancomycin MIC of 1 μg/mL. Since ST398-MSSA has been suggested as the precursor of livestock-associated ST398-MRSA, this indicates that we should pay more attention to the prevalence of such *S. aureus* strains [46]. Most notably, two MR-hVISA strains were SCC*mec* type V, and one PVL-positive MR-hVISA strain was SCC*mec* type IV, and therefore several hVISA and VISA strains might come from the community [47]; the community MRSA clone USA300 with a VISA phenotype, found in San Francisco and Kansas, has been described [48], [49].

Several changes in phenotypes have been described in studies of clinical or laboratory-induced hVISA and VISA strains [8], [21], [37]. In the present study, hVISA and VISA isolates displayed higher prevalence of *agr* dysfunction, reduced adherence ability, and reduced autolytic activity compared to VSSA isolates. We note a strong association between reduced vancomycin susceptibility and *agr* dysfunction, which is similar to previous data [37], [50]. Loss of *agr* function has been associated with the development of vancomycin resistance [37], [44] and prolonged bacteremia [51], but the mechanism underlying reduced levels of *agr* expression in hVISA/VISA strains is not completely understood and requires further study. Interestingly, our data from the biofilm assay showed that adherence ability was reduced in all hVISA and VISA strains compared with that of the VSSA strains. This differs from the report of Sakoulas *et al*. [37] but is similar to the findings of Howden *et al*. [21]. Although biofilm formation differs between laboratory-derived strains and clinical strains [21], the mechanism of altered biofilm formation in clinical strains remains unclear, and further work is needed to understand this.

The results of this study carry several implications. First, clinicians and microbiologists should be aware that all *S. aureus* isolates, obtained from diverse clinical sites, have the potential to develop varying degrees of susceptibility to vancomycin, or to produce subpopulations of mixed susceptibility. Therefore, in future studies we recommend that *S. aureus* isolates from different clinical specimens should be screened for susceptibility to vancomycin. Second, the two-step algorithm we adopted in this study appears to be an improved method for detecting hVISA. By detecting hVISA, clinicians can optimize treatment strategies, reduce associated mortality, shorten the length of stay, and lower patients' hospital charges. Finally, this study should act as a warning of the commonness of hVISA strains. Certainly the finding that hVISA strains are relatively common in Northeast China has alerted us to the need to monitor closely the effect of vancomycin when treating severe *S. aureus* infections. This study may provide incentive for a larger-scale investigation and prevention program, not only in China but also within all international biomedical and epidemiological communities.

We note several limitations of the present study. The period of collecting isolates was short and the sample size was small. Therefore, our report may only hint at the possible prevalence of hVISA and VISA. Furthermore, a recent study found that the MICs of isolates were inversely associated with time in cold storage [52]. In the present retrospective study, all the isolates were recovered from −80°C storage for MIC determination 1 to 4 years after collection, and we cannot guarantee that the MICs were not affected. We also were unable to obtain medical records regarding demographics, underlying diseases, history of exposure to antimicrobials, outcomes, and so forth. However, the results we have reported herein are of sufficient portent to warrant a prospective study of longer duration, with more isolates and complete patient demographic and clinicopathological data.

In summary, this study conducted in Northeast China from 2007 to 2010 shows that VISA strains were rare, but hVISA strains were unexpectedly more common. In addition, combined use of BHIA-3V and PAP-AUC can improve the accuracy of detection and reduce the number of false-positive results. In an effort to prevent the emergence and spread of vancomycin resistance, *S. aureus* isolates from diverse clinical sites should be included when testing for reduced vancomycin susceptibility. Further studies concerning optimal laboratory detection methods and the clinical significance of reduced vancomycin susceptibility in *S. aureus* will be helpful in controlling the development of vancomycin resistance and improving treatment strategies.
